# Metformin is not associated with lactic acidosis in patients with diabetes undergoing coronary artery bypass graft surgery: a case control study

**DOI:** 10.1186/s40360-017-0145-6

**Published:** 2017-05-30

**Authors:** Rakan I. Nazer, Khalid A. Alburikan

**Affiliations:** 1 Department of Cardiac Science, King Fahad Cardiac Center, College of Medicine, King Saud University, KSU 3642, Riyadh, 12372-7143 Kingdom of Saudi Arabia; 2 Department of Clinical Pharmacy, College of Pharmacy, King Saud University, Riyadh, Kingdom of Saudi Arabia

**Keywords:** Metformin, Coronary artery disease, CABG, Lactic acidosis

## Abstract

**Background:**

Metformin associated lactic acidosis (MALA) is a rare but lethal complication. There is no consensus regarding when to stop and resume metformin in patients who undergo coronary artery bypass grafting (CABG). This study aimed to determine if uninterrupted metformin administration in patients with diabetes undergoing CABG increases the risk of lactic acidosis.

**Methods:**

Over a span of 12 months (2015–2016), 127 patients with type 2 diabetes underwent isolated CABG. Of those, 41 patients (32%) continued taking metformin and 86 patients (68%) took other antidiabetic agents. Patients taking metformin took the drug until the day of surgery and resumed taking it 3 h after extubation.

**Results:**

There were no differences in clinical outcomes or complications between groups. Serial measurement of cardiac, liver, and kidney biomarkers were similar between groups. The mean peak lactic acid level was significantly higher in the non-metformin users (5.4 ± 2.6 vs. 7.4 ± 4.1 mmol/l; *P* = 0.001). Multivariable logistic regression analysis identified the need for vasopressor administration as an independent predictor of lactic acidosis (odds ratio: 7.3, 95% confidence interval: 2.5–20.6; *P* < 0.001).

**Conclusion:**

In the absence of risk factors associated with persistent lactic acidosis, such as shock or acute kidney or liver injury, continued peri-operative metformin administration was not associated with the occurrence of lactic acidosis in patients undergoing CABG. Elevated lactic acid levels seem to be directly related to tissue anoxia caused by escalating vasopressor support after surgery.

## Background

Metformin is an oral antidiabetic agent used as a first line intervention in the treatment of type 2 diabetes. Belonging to the biguanide group of hypoglycemics, metformin mainly acts by suppressing gluconeogenesis in the liver, decreasing glucose absorption in the digestive tract, and increasing muscle and fatty tissue sensitivity to insulin [1]. Metformin associated lactic acidosis (MALA) is a rare but fatal condition with a reported incidence of 3.3–9.7 cases per 100 000 patient-years and a mortality rate of up to 45% [2, 3]. The accumulation of lactate is in part due to the inhibition of the respiratory chain in the mitochondrial membrane, leading to decreased uptake of pyruvate, which acts as a precursor to lactate in gluconeogenesis [4]. Independent from its glucose lowering effect, metformin monotherapy has been linked to weight reduction, improved survival, reduction of myocardial infarction size, reduced risk of infection, and a significant reduction in overall cardiovascular morbidity [5–9]. Currently, there are no existing safety guidelines concerning metformin administration during the perioperative period in patients scheduled for surgery. Some suggest discontinuing metformin therapy 48 h prior to surgery [10, 11]. Others see no reason to discontinue its use unless other major risks for lactic acidosis co-exist, such as renal failure, liver failure, respiratory failure, and shock [7, 12].

The objective of this study was to determine whether continuing perioperative metformin administration in patients with diabetes during coronary artery bypass grafting (CABG) leads to significant lactate accumulation when compared to patients with diabetes who are not treated with metformin.

## Methods

This study was approved by our institutional research ethics committee and conformed to the tenets of the Declaration of Helsinki, and the electronic registry for all cardiac procedures performed at King Fahad Cardiac Center was interrogated for isolated on-pump CABG from the beginning of December 2014 up to the beginning of January 2016 (12 months). One month prior to this interval, we adopted the policy of continued administration of metformin up to the day of surgery and its early resumption soon after extubation (within 2–3 h) in patients with diabetes who were originally on this drug, in the absence of contraindications or other risk factors for lactic acidosis. Contraindications to metformin therapy were defined as follows: renal dysfunction (creatinine clearance less than or equal to 60 ml/min) and receiving two or more nephrotoxic medications. A total of 193 patients undergoing isolated on-pump CABG were identified. Of those, 127 patients (65%) had type 2 diabetes mellitus. In this subset, 41 patients (32%) were on long-term metformin therapy (mean duration, 26 ± 9 months). The remaining 86 patients (68%) were on other hypoglycemic agents. Home insulin was used by 24 patients (20%); of those, 6 patients (5%) used insulin combined with metformin. The other 18 patients (14%) on insulin therapy used either insulin alone or combined with other non-metformin antidiabetic agents. Patients who used metformin were labeled as group I and those who did not use metformin were labeled as group II. Individual patient consent was waived and all relevant demographic, clinical, and laboratory data were retrospectively collected from the patients’ charts, intensive care unit (ICU) flow sheets, and electronic laboratory records. Serum lactic acid was measured on an hourly basis in the ICU during blood gases analysis, and the peak lactic acid (mmol/L) within the first 24 h after surgery was recorded for every patient in the study. Similarly, the lowest bicarbonate (HCO_3_) (mmol/L) and peak carbon dioxide (PCO_2_) (mmHg) were recorded from blood gas analysis in the first 24 h after surgery. Cardiac enzymes total creatinine kinase [total CK (U/L)], creatinine kinase MB fraction [CK-MB (U/L)], and troponin I (ug/L) were measured daily for first two days after surgery, and the peak level of each enzyme was recorded for every patient in the study. Fasting blood glucose (mmol/L), serum creatinine (umol/L), total bilirubin (mmol/L), alkaline phosphatase (ALP) (U/L), alanine aminotransferase (ALT) (U/L), aspirate aminotransferase (AST) (U/L), gamma-glutamyltransferase (GGT) (U/L), and lactate dehydrogenase (LDH) (U/L) were measured prior to surgery and daily for up to 7 days after surgery. Post-operative fasting glucose was recorded as the mean level after surgery and the peak level of the remaining enzymes was recorded for each patient after surgery. Acute kidney injury after surgery was defined as a greater than 50% increase in baseline serum creatinine. Post-operative lactic acidosis was defined as an elevation of serum lactate to 5 mmol/l or greater with a bicarbonate level less than 22 mmol/l on blood gas analysis [13].

All patients underwent CABG under general anesthesia with full heparinization and central cannulation of the aorta and right atrium. The heart was arrested with cold blood potassium cardioplegia. All patients received a left internal mammary graft on the left anterior descending coronary artery with a segment of the greater saphenous vein on the other coronary targets. Intraoperatively, blood glucose was measured every 30 min along with activated clotting time (ACT), electrolytes, and blood gases. Once the patient arrived in the ICU, blood glucose was measured every hour. All patients were subjected to a hospital-wide standardized glycemic control protocol using continuous regular insulin infusion. The aim of therapy was to maintain blood glucose between 5.6–8.3 mmol/l in the operating room and ICU. If blood glucose exceeded 13.8 mmol/l, then added boluses of regular insulin were administered in accordance with a sliding scale. When blood glucose reached 3.3–5.5 mmol/l, insulin infusion was reduced by 50%. When blood glucose was < 3.3 mmol/l, insulin infusion was stopped and 50 ml of 50% dextrose bolus was administered. After surgery, all patients spent at least 24 h in the ICU; thereafter they were transferred to a monitored bed in the cardiac ward until the day of discharge. Patients on metformin prior to surgery received the following dosages: 500 mg (72%), 850 mg (20%), and 1,000 mg (8%) every 12 h.

Statistical analysis was performed using SPSS 16.0 software (SPSS Inc., Chicago, IL). Categorical data were tabulated in 2 × n tables and two-group comparisons were performed using the *chi-square* test or Fisher exact test. Continuous variables were expressed as means ± standard deviations. The t test was used to compare two-group continuous variables. Variables deemed important by univariable analysis *(P <* 0.2) or those deemed clinically significant were entered into a multivariable logistic regression model. The results are expressed as odds ratios (OR), 95% confidence interval (95% CI), and *P* values. A two-tailed value of <0.05 was considered to be significant for all statistical tests.

## Results

**Fig. 1:**
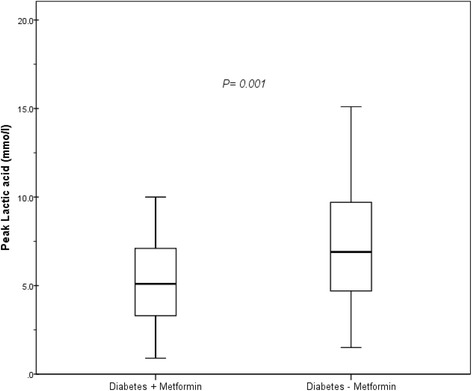
Comparison of peak serum lactic acid levels in the first 24 h after surgery in the two patient groups. (Patients with diabetes who took metformin perioperatively = Diabetes + metformin, patients with diabetes were not on metformin = Diabetes - metformin)

**Fig. 2:**
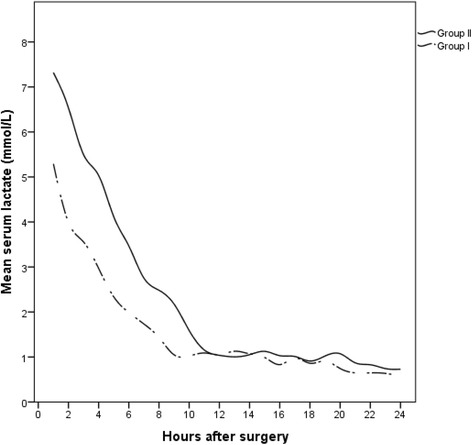
Linear trend of the mean lactic acid levels in the first 24 h after surgery (Patients with diabetes who took metformin perioperatively = Group I, patients with diabetes who were not on metformin = Group II)

For both groups (group I: metformin users, group II: other hypoglycemic users), demographic characteristics including age, sex, body mass index (BMI), history of acute myocardial infarction, ventricular function, and symptoms were similar. The calculated operative mortality risks according to European System for Cardiac Operative Risk Evaluation (EURO II ) for the two groups were statistically similar (group I: 1.08 ± 0.1, group II: 1.29 ± 0.1, *P* = 0.257; Table 1). Glycosylated hemoglobin A1C % (HbA1C) was significantly higher in group I (9.0 ± 1.5 vs. 8.1 ± 1.6; *P* = 0.010). Perioperatively, there were no significant differences in the cardiopulmonary bypass or cross-clamp times (103.5 ± 32.3 vs. 100.8 ± 38.9 min, *P* = 0.709 and 83.2 ± 31.3 vs. 79.8 ± 32.3, *P* = 0.590, respectively; Table 2). The peak blood glucose values recorded intraoperatively were significantly higher for group I (11.5 ± 2.6 vs. 10.4 ± 3.1 mmol/L; *P* = 0.034). The immediate postoperative course was similar for both groups. The mean cardiac index in group I and group II was 3.0 ± 0.5 vs. 2.9 ± 0.5 L/M^2^/min (*P* = 0.277) and the time spent on the ventilator was 8.0 ± 15.0 vs. 8.0 ± 9.2 h (*P* = 0.998), respectively. When comparing the post-operative laboratory measurements (Table 3), fasting blood glucose was significantly higher in group I (10.0 ± 1.3 vs. 8.9 ± 2.1 mmol/l; *P* = 0.001). Interestingly, the need for more than one vasopressor in the immediate postoperative period was significantly higher in group II (12.2% vs. 27.9%; *P* = 0.049). There were no significant differences in the peak levels of cardiac enzymes, creatinine, and liver enzymes (Table 3). Other clinical outcomes revealed a trend for lower rates of myocardial infarction, atrial fibrillation, low output state, and surgical site infection in group I, but these differences did not reach statistical significance. Two patients died perioperatively (0% in group I vs. 2.3% in group II; *P* = 1.0); deaths were due to perioperative myocardial infarction and post-cardiotomy shock. The mean peak lactic acid level (Table 3) was significantly higher in group II (5.4 ± 2.6 vs. 7.4 ± 4.1 mmol/l; *P* = 0.001; Fig. 1) as was the mean post-operative lactic acid in the first 24 h after surgery (3.1 ± 3.2 vs. 5.7 ± 4.4 mmol/l; *P* = 0.001; Fig. 2). Lactic acid levels peaked for both groups within the first 2 h after surgery, and thereafter reduced to baseline 8–10 h after surgery (around the time of extubation). There was no difference between the two groups in perioperative peak PCO_2_ (41.3 ± 3.5 vs. 40.4 ± 4.5 mmHg; *P =* 0.235). Peak LDH was also higher in group II (414.0 ± 159.8 vs. 543.7 ± 337.2 U/l; *P* = 0.021). There were also more patients in group II who developed transient post-operative lactic acidosis (arterial lactate level > or = 5 mmol/L and serum bicarbonate of < or = 22 mmol/l) (23.8% vs. 76.2%, *P* = 0.043). A multivariable logistic regression model was used to evaluate the following factors: age >70 years, elevated post-operative creatinine > 50% of baseline, cardiac low output state, continued perioperative metformin, and the requirement for vasopressors in the ICU. This analysis revealed that additional vasopressor use was an independent predictor for transient post-operative lactic acidosis (OR: 7.26, 95% CI: 2.55–20.65; *P* < 0.0001) within the first 24 h after CABG (Table 4).

**Table 1:** Baseline characteristics

	Group I Diabetes + metformin *n* = 41 (32%)	Group II Diabetes - metformin *n* = 86 (68%)	*P*-value
Age (years) ± SD	57.3 ± 9.2	57.2 ± 10.0	0.969
Female (%)	7 (17.1%)	11 (12.8%)	0.518
Body mass index (BMI)	27.6 ± 5.2	27.8 ± 5.6	0.832
Myocardial infarction (STEMI/NSTEMI)	19 (46.3%)	51 (59.3%)	0.170
Urgent CABG	6 (14.6%)	18 (20.9%)	0.397
Post-op stay (days) ± SD	7.3 ± 7.2	8.7 ± 19.6	0.658
Median post-op stay (days)	5.0	5.0	
New York Heart Association Class III-IV (%)	12 (29.3%)	21 (24.4%)	0.560
Canadian Cardiac Society Class III-IV (%)	28 (68.3%)	44 (51.2%)	0.069
Ejection fraction <40 (%)	9 (22.0%)	14 (16.3%)	0.438
EuroSCORE II	1.08 ± 0.1	1.29 ± 0.1	0.257
Redo-CABG	0	0	
Risk factors and comorbidities			
Insulin injection (%)	6 (14.6%)	18 (20.9%)	0.397
Glycosylated Hemoglobin A1C (%)	9.0 ± 1.5	8.1 ± 1.6	0.010
Total cholesterol-HDL ratio	4.8 ± 2.9	5.0 ± 1.7	0.729
Baseline serum creatinine (umol/l)	87.0 ± 22.0	93.0 ± 22.8	0.165
Blood sugar (mmol/l)	11.0 ± 4.3	10.8 ± 18.9	0.950
Hypertension (%)	36 (87.8%)	68 (79.1%)	0.232
Hyperlipidemia (%)	27 (65.9%)	45 (52.3%)	0.150
Positive family history (%)	8 (19.5%)	18 (20.9%)	0.853
Current smoking (%)	7 (17.1%)	23 (26.7%)	0.230
Chronic lung disease (%)	0 (0%)	6 (7.0%)	0.176
Stroke (%)	3 (7.3%)	5 (5.8%)	0.712
Peripheral vascular disease (%)	1 (2.4%)	11 (12.8%)	0.101
Elevated creatinine >140 umol/l (%)	1 (2.4%)	5 (5.8%)	0.663
Significant carotid disease (%)	0 (0%)	4 (4.7%)	0.304
Viral hepatitis (%)	1 (2.4%)	1 (1.2%)	0.543

**Table 2:** Peri-operative outcomes

	Group I Diabetes + Metformin *n* = 41 (32%)	Group II Diabetes - Metformin *n* = 86 (68%)	*P*-value
Ventilation time (hours) ± SD	8.0 ± 15.0	8.0 ± 9.2	0.998
Intensive care time (hours) ± SD	42.8 ± 33.7	46.1 ± 45.4	0.692
Cardiopulmonary bypass time (minutes) ± SD	103.5 ± 32.3	100.8 ± 38.9	0.709
Cross clamp time (minutes) ± SD	83.2 ± 31.3	79.8 ± 32.3	0.590
Post-op cardiac index (mean) ± SD	3.0 ± 0.5	2.9 ± 0.5	0.277
Lowest mixed venous oxygen saturation % (SVO2)	55.8 ± 6.4	55.9 ± 8.6	0.950
Number of vasopressors >1	5 (12.2%)	24 (27.9%)	0.049
Intra-aortic balloon pump (%)	2 (4.9%)	8 (9.3%)	0.498
Myocardial infarction (%)	1 (2.4%)	6 (7.0%)	0.427
Low output syndrome (%)	7 (17.1%)	21 (24.4%)	0.351
New post-op dialysis (%)	0 (0%)	1 (1.2%)	1.000
Atrial Fibrillation (%)	0 (0%)	7 (8.1%)	0.095
Pulmonary complications (%)	7 (17.1%)	19 (22.1%)	0.640
Stroke/Transient ischemic attack (%)	1 (2.4%)	3 (3.5%)	1.000
Surgical site infection (%)	2 (4.9%)	7 (8.1%)	0.717
Death (%)	0 (0%)	2 (2.3%)	1.000

**Table 3:** Peri- operative laboratory measurements

	Group I DM + Meformin *n* = 41 (32%)	Group II DM - Metformin *n* = 86 (68%)	*P*-value
Serum glucose			
^a^Fasting blood sugar (mmol/l) ± SD	10.0 ± 1.3	8.9 ± 2.1	0.001
Cardiac enzymes			
^b^Peak CK (U/l) ± SD	1128.5 ± 1065.1	1062.6 ± 772.8	0.693
^b^Peak CK-MB (U/l) ± SD	86.4 ± 126.9	82.3 ± 66.9	0.809
^b^Peak troponin I (ug/l) ± SD	2.1 ± 3.1	3.3 ± 4.8	0.185
Kidney			
^b^Peak creatinine (mmol/l) ± SD	109.7 ± 25.9	119.8 ± 37.2	0.122
^c^Acute kidney injury	7 (17.1%)	17 (19.8%)	0.717
Liver			
^b^Peak total bilirubin (mmol/l) ± SD	17.6 ± 15.5	21.1 ± 15.7	0.244
^b^Peak ALP (U/l) ± SD	119.7 ± 57.9	135.9 ± 92.7	0.307
^b^Peak ALT (U/l) ± SD	71.0 ± 85.1	124.6 ± 251.3	0.079
^b^Peak AST (U/l) ± SD	88.0 ± 108.1	134.7 ± 278.6	0.303
^b^Peak GGT (U/l) ± SD	99.0 ± 90.8	107.9 ± 120.7	0.677
Other laboratory measures			
^b^Peak LDH (U/l) ± SD	414.0 ± 159.8	543.7 ± 337.2	0.021
^b^Peak lactic acid (mmol/l) ± SD	5.4 ± 2.6	7.4 ± 4.1	0.001
Mean lactic acid (mmol/l) ± SD	3.1 ± 3.2	5.7 ± 4.4	0.001
^d^Transient lactic acidosis (%)	15 (23.8%)	48 (76.2%)	0.043
^b^Peak PCO2 (mmHg)) ± SD	41.3 ± 3.5	40.4 ± 4.5	0.235
^e^Lowest HCO3 (mmol/l) ± SD	20.1 ± 1.8	18.9 ± 2.8	0.008

^a^The mean post-operative fasting blood glucose

^b^The mean of the highest reading in the peri-operative period ± standard deviation

^c^Elevation of serum creatinine > 50% of baseline measurement

^d^The patients with transient post-operative lactic acid > 5 U/l and bicarbonate < 22 mmol/l

^e^The mean of the lowest reading in the peri-operative period ± standard deviation

**Table 4:** Logistic regression predictors for peri-operative lactic acidosis

Variables	Univariable analysis	Multivariable analysis
Age >70	OR: 1.53; 95%CI 0.75–3.12, *P* = 0.237	OR: 2.12; 95%CI 0.93–4.83, *P* = 0.073
Elevated serum creatinine >50%	OR: 1.01; 95%CI 0.19–5.2, *P =* 0.984	OR: 1.69; 95%CI 0.23–12.30, *P* = 0.601
Peri-operative metformin	OR: 0.45; 95%CI 0.21–0.98, *P* = 0.045	OR: 1.78; 95%CI 0.77–4.09, *P* = 0.174
Cardiac low out-put state	OR: 2.16; 95%CI 0.91–5.15, *P* = 0.082	OR: 1.74; 95%CI 0.49–6.17, *P* = 0.393
Vassopressor support	OR: 7.26; 95%CI 2.55–20.65, *P* < 0.0001	OR: 11.77; 95%CI 2.86–48.37, *P* < 0.001

## Discussion

Combining metformin with insulin in non-cardiac surgical ICU patients is associated with better glucose control without the occurrence of lactic acidosis [14–16]. The intention of this study was to determine the incidence of adverse events related to the early administration of metformin after CABG. As with earlier studies, our investigation did not indicate that metformin was associated with a significant increase in lactic acid levels when compared to patients who were not taking metformin for diabetes. In fact, in a systemic review by Salpeter et al. [4] evaluating pooled data from 347 comparative trials comparing the incidence of lactic acidosis in patients with diabetes who took metformin versus those who did not, a lower incidence of lactic acidosis was found in the metformin group (0.0043% vs. 0.0054%). However, no cardiac surgery patients were included in this analysis. A similar finding was reported by Stang et al. [17] Similarly, our cohort study shows a smaller peak in serum lactate in patients who used metformin early after CABG (5.4 ± 2.6 vs. 7.4 ± 4.1; *P* = 0.001), which was associated with a significantly higher bicarbonate level (20.1 ± 1.8 vs. 18.9 ± 2.8; *P* = 0.008). The occurrence of lactic acidosis with metformin ingestion is rare and sporadic [18]. The majority of patients who developed lactic acidosis usually have other serious underlying conditions such as shock, sepsis, or acute kidney or liver disease. In both animal and human models, hypothermic cardio-pulmonary bypass was associated with subtle hemodynamic changes in the mucosal and hepatic sinusoidal microcirculations, which add to splanchnic hypoperfusion and ischemia [19]. This is further aggravated by the post-operative increased oxygen demand, low output cardiac state, and use of vasoactive agents. This cascade of events can inflect visceral organ injury manifesting in a recognized pattern of liver, kidney, and pancreas biomarker surge within the first 2–3 days after surgery [20–22].

Metformin use in patients with diabetes undergoing cardiac surgery was not shown to increase the risk for adverse cardiac outcomes. Duncan et al. [7] retrospectively matched 443 patients who took metformin up to 8 h prior to cardiac surgery with the same number of non-metformin treated patients using propensity scores. There was no difference in major adverse events between the two groups. In fact, the metformin users had overall less morbidities (OR (95% CI) 0.4 (0.2–0.8); *P* = 0.005), leading the authors to suggest that pre-treatment with metformin might have beneficial effects. In a randomized clinical trial, Baradari et al. [23] randomized 200 patients with diabetes to either high dose metformin (1,000 mg) twice daily versus placebo within 3 h of extubation after CABG. There were no differences in the serum lactate levels during the 5 consecutive days after surgery and there was no acidosis in either group. Another prospective study done by the same group found that adding metformin to insulin regimens led to better glycemic control in patients with diabetes who undergo CABG without causing metabolic acidosis [24]. It is not clear in both of these studies whether metformin was continued up until the day of surgery or stopped a few days prior. El Messaoudi et al. [25] randomized 111 patients without diabetes to 500 mg metformin three times a day versus placebo 3 days prior to elective on-pump CABG. Similarly, there was no increase in lactic acidosis or adverse events in the metformin group. In the current study, all patients in group I received metformin up until the day of surgery and resumed taking it 2–3 h post extubation. We observed a trend towards more peri-operative myocardial infarction, atrial fibrillation, and infection in group II, which might explain the need for additional vasopressors in the ICU. Epinephrine and other vasoconstrictive agents may cause vasoconstriction and elevation in serum lactate following cardiopulmonary bypass. In a study by Jabbari et al. [26], 15 patients had their lactic acid level measured in the operating room and ICU. The average lactate was initially 0.83 ± 0.28 mmol/l on induction for surgery, 0.87 ± 0.33 a half hour from induction, 2.84 ± 1.68 after a quarter of an hour on cardio-pulmonary bypass, 3.48 ± 2.23 at the end of cardio-pulmonary bypass, 4.28 ± 2.48 at the end of surgery, and 4.33 ± 2.56 during ICU admission. A similar study by Meng et al. [22] measured lactate and other inflammatory markers in patients undergoing on-pump CABG every 6 h for 24 h after the start of cardio-pulmonary bypass. The lactate was recorded as follows: T0 (1.12 ± 1 mmol/l), T1 (1.15 ± 0.07), T2 (3.12 ± 0.21), T3 (2.97 ± 0.18), T4 (2.58 ± 0.25). Our current study has identified the use of vasopressors in the peri-operative period as an independent predictor of lactic acidosis. Other authors have identified increased lactate concentration after on-pump CABG to be associated with total inotrope dosage, hemodynamic parameters, duration of pump time, and cross-clamp time [26].

The current study is limited by its retrospective nature, its small sample size, and variability in metformin dosage; however, it confirms the same findings reported in other studies [18, 23, 24, 26, 27].

## Conclusion

Our results show that continuing metformin administration in the peri-operative period is unlikely to cause lactic acidosis or other major adverse events. Evidence from the literature indicates that metformin use in patients with diabetes after CABG surgery tends to be associated with better glucose control and reduced complications.

## Abbreviations

95% CI: 95% confidence interval
ACT: Activated clotting time
ALP: Alkaline phosphatase
ALT: Alanine aminotransferase
AST: Aspirate aminotransferase
BMI: Body mass index
CABG: Coronary artery bypass grafting
Euro II score: European System for Cardiac Operative Risk Evaluation Score
GGT: Gamma-glutamyltransferase
HbA1C: Glycosylated hemoglobin A1C %
HCO3: Bicarbonate
ICU: Intensive care unit
LDH: Lactate dehydrogenase
MALA: Metformin associated lactic acidosis
OR: Odds ratios
PCO2: Peak carbon dioxide
